# Trehalose Rescues Postmenopausal Osteoporosis Induced by Ovariectomy through Alleviating Osteoblast Pyroptosis via Promoting Autophagy

**DOI:** 10.3390/biomedicines12102224

**Published:** 2024-09-29

**Authors:** Xinli Hu, Wei Wang, Xiaolong Chen, Chao Kong, Xuan Zhao, Zheng Wang, Haojie Zhang, Shibao Lu

**Affiliations:** 1 Department of Orthopedics, Xuanwu Hospital, Capital Medical University, No. 45 Changchun Street, Xicheng District, Beijing 100053, China; wangwei37@buaa.edu.cn (W.W.); chensmalldragon@163.com (X.C.); kong98850@163.com (C.K.); zxc911@126.com (X.Z.); spinewzccmu@163.com (Z.W.); haojiezhang@163.com (H.Z.); shibaolu@ccmu.edu.cn (S.L.); 2 National Clinical Research Center for Geriatric Diseases, Xuanwu Hospital, Capital Medical University, No. 45 Changchun Street, Xicheng District, Beijing 100053, China

**Keywords:** autophagy, inflammation, osteoporosis, pyroptosis, trehalose

## Abstract

Background: Osteoporosis, a prevalent bone metabolic disease, often requires long-term drug treatments that may lead to serious side effects. Trehalose, a natural disaccharide found in various organisms, has been shown to have a promoting effect on autophagy. However, whether trehalose can improve bone mass recovery in ovariectomized rats and its underlying mechanisms remains unclear. In this study, trehalose was administered to ovariectomized rats to evaluate its therapeutic potential for osteoporosis following ovariectomy. Methods: Micro-computed tomography (Micro-CT), hematoxylin and eosin (HE) and immunohistochemical staining techniques were utilized to evaluate the impact of trehalose on osteoporosis induced by ovariectomy (OVX) in mice, both in imaging and histological dimensions. Furthermore, the influence of trehalose on osteoblastogenesis and functional activity was quantified through Alizarin Red S (ARS) staining and immunoblotting assays. Results: Trehalose effectively mitigated bone loss, elevated autophagy and suppressed pyroptosis in ovariectomized rats. Furthermore, 3-methyladenine diminished the protective effects of trehalose, particularly in promoting autophagy and inhibiting pyroptosis. Conclusions: Trehalose demonstrates significant potential in treating osteoporosis by suppressing NLRP3 inflammasome-driven pyroptosis, primarily through autophagy promotion. This suggests that trehalose could be a promising, safer alternative treatment for osteoporosis.

## 1. Introduction

Osteoporosis is a skeletal disorder predominantly observed in the elderly, and the main clinical manifestations include diminished bone mineral density (BMD), exacerbated bone microarchitecture, and enhanced susceptibility to fractures and bone fragility [1]. As a significant public health challenge, it is strongly correlated with increased disability, morbidity, mortality, and substantial healthcare expenditures [2]. The pathology of the disease originates from a dysregulation in the dynamic equilibrium between bone formation and bone resorption [3]. Crucially, heightened osteoblast cell death and diminished osteogenic differentiation are its primary pathological hallmarks [4]. Hence, promoting osteoblast survival and bolstering osteoblastic functions are vital for restoring bone equilibrium, making them central to effective osteoporosis management.

Pyroptosis is an inflammation-based programmed cell death (PCD) with significant implications in osteoporosis development, primarily through inflammasome activation [5,6]. The inflammasome complex comprises three key components: the NOD-like receptor, the cysteine protease caspase-1, and the adaptor protein known as apoptosis-associated speck-like protein with a CARD (ASC) [7,8]. NLRP1, NLPR3, NLRC4, pyrin, absent in melanoma 2 (AIM2) and other inflammasomes have been identified, with NLRP3 being the most extensively studied [9]. NLRP3 inflammasomes can detect PAMPs (pathogen-associated molecular patterns) or DAMPs (damage-associated molecular patterns), subsequently recruiting and activating the pro-inflammatory protease caspase-1. Upon activation, caspase-1 facilitates the cleavage of pro-IL-1β/18 and GSDMD (Gasdermin D) [10]. The resulting GSDMD-N creates 10–15 nm pores in the cell membrane, culminating in the release of IL-1β/18 [11]. Numerous studies indicate that pyroptosis is intricately linked to various metabolic diseases that are exacerbated by aging and chronic inflammation [12,13,14]. Furthermore, osteoporosis involves an abundance of inflammatory factors and signaling pathways, suggesting a correlation between inflammation and osteoporosis onset [15,16]. Several findings also suggest that pyroptosis can impact osteoblast and osteoclast functionality, contributing to osteoporosis [5,17]. Thus, strategies targeting pyroptosis offer a promising avenue for osteoporosis treatment.

Autophagy is an essential intracellular degradation mechanism that mediates the transport of cytosolic components, such as longevity proteins, misfolded proteins, and defective or superfluous organelles, to lysosomes for breakdown via double-membraned autophagosomes [18]. Growing evidence underscores the role of autophagy in supporting the survival and function of osteoblasts, osteocytes, and osteoclasts [19,20]. Notably, several studies have illustrated how autophagy aids in the removal of NLRP3 inflammasome activators, its components, and cytokines, ultimately leading to reduced pyroptosis and decreased inflammatory cytokine release across various disease conditions [21,22,23]. Given these insights, there is a prevailing hypothesis among researchers that autophagy might regulate osteoblast death in osteoporosis by curtailing pyroptosis. As such, there is an emergent need to discover drugs that can inhibit pyroptosis by harnessing the power of autophagy.

Trehalose is a natural disaccharide existing in organisms of plants, bacteria, yeast, and fungi, etc. It has emerged as a promising autophagy activator in multiple cells, operating via an mTOR-independent pathway [24,25]. Beyond its role in autophagy, trehalose has displayed protective effects against cellular stresses, including oxidative damage, dehydration, and temperature fluctuations [26]. Recent research suggests that trehalose can counter osteoporosis, attributed to its ability to inhibit collagen degradation [27]. Particularly, according to some studies, trehalose can counter osteoporosis by enhancing AKT/TFEB pathway-dependent autophagy flow [28]. In another study, trehalose enhanced bone mass in rats with cirrhosis by promoting autophagosome formation, hinting at its potential function in osteoporosis treatment [29]. However, the role of trehalose in mitigating pyroptosis in osteoporosis has not been thoroughly investigated. It remains uncertain whether trehalose inhibits pyroptosis by stimulating autophagy. Considering this, our study delves deeper into how trehalose affects autophagy and pyroptosis, assessing its efficacy in restoring bone mass in osteoporotic rats. We hypothesize that trehalose can inhibit estrogen deficiency-induced pyroptosis through autophagy activation, and that the therapeutic effects of trehalose can be reversed by inhibiting autophagy with 3-MA, which is a classical autophagy inhibitor. This study provides new molecular targets for the treatment of osteoporosis with trehalose and may offer evidence for its potential therapeutic use in postmenopausal osteoporosis.

## 2. Materials and Methods

### 2.1. Chemicals and Reagents

D-(+)-Trehalose dehydrate, with a purity exceeding 99%, was sourced from Sigma-Aldrich (St. Louis, MO, USA). α-MEM, fetal bovine serum (FBS), and phosphate-buffered saline (PBS) were procured from Servicebio (Wuhan, China). 3-Methyladenine (3 MA), characterized by a molecular formula of C6H7N5 and a high-performance liquid chromatography (HPLC) purity of ≥99% (cat# M9281), came from Sigma-Aldrich. Histological staining included hematoxylin-eosin (HE) staining (cat# G1120) and Masson’s trichrome staining (cat# G1340) (Solarbio Science & Technologies, Beijing, China). The investigation employed primary antibodies including Beclin1 (Cell Signaling Technology, cat# 3738, Danvers, MA, USA), LC3 (Cell Signaling Technology, cat# 3868), Cathepsin D (CTSD) (Proteintech, cat# 21327-1-AP, Rosemont, IL, USA), VPS34 (Proteintech, cat#12452-1-AP), p62 (Abcam, cat# ab56416, Hong Kong, China), NLRP3 (Cell Signaling Technology, cat#15101), caspase-1 (Proteintech, cat#22915-1-AP), ASC (Cell Signaling Technology, cat# 67824), GSDMD (Affinity, cat# AF4013, West Bridgford, UK), IL-1β (ABclonal, cat# A11369, Swansea, UK), IL-18 (Affinity, cat# DF6252), and GAPDH (Proteintech, cat#10494-1-AP). Secondary antibodies used included Goat anti-rabbit IgG (H+L)-HRP (Bioworld, cat# BS13278, Dublin, OH, USA) and Goat anti-mouse IgG(H+L)-HRP (Bioworld, cat# BS12478).

### 2.2. Animals

Obtained from the Laboratory Animal Center of Xuanwu Hospital, Capital Medical University, female Sprague Dawley (SD) rats (age: 8 weeks; average weight: 200 ± 20 g) were housed under controlled environmental conditions, including 50–60% relative humidity, an ambient temperature of 20 ± 2 °C, and a 12 h light/dark cycle, with free access to water and food. All experimental protocols involving animals obtained the approval of the Ethics Committee of Capital Medical University (approval number: SYXK (Jing) 2021-0008) and adhered to the Institutional Animal Care and Use Committee (IACUC) guidelines of Capital Medical University. The experimental procedures also followed the guidelines of the NIH publication “Care and Use of Laboratory Animals” (1996).

### 2.3. Experiment Design

After a one-week acclimation period, seventy female rats fell into 7 groups in a random manner (n = 10 per group): Sham group, OVX group, OVX + low-dose trehalose group (1 mg/kg), OVX + high-dose trehalose group (2 mg/kg), OVX + E2 group (estrogen), OVX + 3 MA group, and OVX + high-dose trehalose + 3 MA group. Anesthesia was induced with 1% sodium pentobarbital (50 mg/kg) prior to surgery, and penicillin was administered for three days post-operation to minimize infection risk. For the trehalose + 3 MA groups, 3 MA (15 mg/kg) was injected 30 min before trehalose administration. The dosage and timing of trehalose administration followed a previously established protocol. OVX rats were treated with trehalose or estrogen via intraperitoneal injection at the designated dosages for 12 weeks. The Sham and OVX groups were subjected to the intraperitoneal injection of normal saline as controls. Before the treatment completed, all rats were weighed and subsequently euthanized. The tibiae and femora from both sides were harvested for histological analysis.

### 2.4. Micro-CT Bone Analysis

Tibia specimens underwent two days of fixation in 4% paraformaldehyde (PFA), followed by meticulous removal of the surrounding soft tissue. Micro-computed tomography (micro-CT) examinations (Skyscan, Bruker, Belgium) and subsequent evaluations of the tibias were executed using the NRecon Reconstruction software (Bruker microCT, Kontich, Belgium). The scans were acquired with a voxel resolution of 9 microns, deploying a source voltage of 100 kV and a source current of 220 μA. Two-dimensional reconstructions were formulated using DataViewer software (Bruker microCT, version number 3.9), whereas three-dimensional visualizations were facilitated by the CTVox software (Bruker microCT, version number 3.9). The tibia’s region of interest (ROI) was demarcated commencing 0.5 mm from the proximal tibial growth plate and spanning to the uppermost region of the tibial cortical bone. Quantitative analyses were undertaken using the these software, enabling the determination of parameters like trabecular bone volume fraction (BV/TV), trabecular number (Tb.N), trabecular thickness (Tb.Th), and trabecular separation (Tb.Sp).

### 2.5. HE and Masson Staining and Immunohistochemistry (IHC)

Tibial bones from rats were fixed in 4% PFA for a duration of 48 h, subsequently demineralized, and then incubated in 15% EDTA solution for a period of 50 days. After this, the bone specimens were subjected to a graded ethanol dehydration process, clarified using xylene for a span of 2 h, embedded within 4% paraffin, and sectioned to produce slices of 5 μm thickness. These sections, post deparaffinization and rehydration received HE and Masson staining based on established methodologies. From each bone specimen, three indicative tibial sections were chosen at random. A Nikon ECLIPSE Ts2 microscope (Tokyo, Japan) was employed for the visualization of stained sections and image capturing, together with the assembling of representative images. Quantitative assessment of bone parameters, such as the trabecular and tissue areas, was executed using the ImageJ software (version number v1.8.0).

For IHC analysis, the preparation of the tibial specimens mirrored the procedure outlined above. Following preparation, these sections received 12 h of incubation by primary antibodies targeting NLRP3 and caspase 1. After incubation, the sections underwent a 15 min wash with PBS before being exposed to their respective biotinylated secondary antibodies. The final staining was performed using diaminobenzidine (DAB; Sigma-Aldrich), with the bone matrix receiving a counterstain using hematoxylin (ACMEC, Shanghai, China).

### 2.6. Immunofluorescence Staining

For immunofluorescence analysis, the tibial specimens were processed in a manner analogous to the aforementioned procedure. The slides were exposed to primary antibodies against p62 and Beclin1 and incubated at 4 °C overnight. Subsequent visualization of these slides was achieved through fluorescence microscopy (Nikon, A1 PLUS, Tokyo, Japan). For the purpose of quantification, a minimum of three distinct fields from each section were selected, and the percentage area of cells exhibiting positive staining was assessed.

### 2.7. Cell Culture and Treatment

Isolated from the femurs and tibias of healthy 2-week-old SD rats, bone marrow stromal cells (BMSCs) were put in α-Minimum Essential Medium (α-MEM, HyClone, Logan, UT, USA) supplemented with 10% FBS and 1% penicillin/streptomycin for culture. The controlled conditions included humidified environment with 5% CO_2_, a temperature of 37 °C, and medium being changed each two days. BMSCs reaching 80–90% confluence were harvested using 0.25% trypsin and sub-cultured. For the experiments, cells from passages 3 to 6 were utilized.

### 2.8. Cell Viability Assessment

A CCK-8 assay kit (Elascience, Wuhan, China) was employed to evaluate cell viability in accordance with the provided protocol. BMSCs were treated as prescribed for 24 h, followed by a replacement of the culture medium with a CCK-8 solution, comprising 10% CCK-8 reagent. The cells then underwent 1 h of incubation at 37 °C. Subsequently, a microplate reader measured the absorbance at 450 nm (Thermo Fisher Science, Waltham, MA, USA).

### 2.9. ELISA Assay

Culture supernatants were harvested, and the concentrations of interleukin-1β (IL-1β) and interleukin-18 (IL-18) were quantified using an enzyme-linked immunosorbent assay (ELISA) kit (Elabscience, Wuhan, China) following the manufacturer’s guidelines.

### 2.10. Alizarin Red Staining (ARS)

BMSCs underwent 3 days of culture on glass slides. Subsequently, the standard culture medium was superseded by osteogenic medium encompassing DMEM added with 0.1 μM dexamethasone, 50 μM ascorbic acid, 10 mM β-glycerol phosphate, and 10% FBS. Post 7 days of osteogenic induction, cells successively received 3 rounds of washing in PBS, 30 min of fixation in 4% PFA at 4 °C, and 20 min of incubation in a BCIP/NBT solution (Beyotime, Nantong, China) in a dark environment. On day 21 of the osteogenic culture regimen, the cells adhered to the glass slides received 15 min of fixation in 95% ethanol. This was followed by a 30 min staining process using 40 mM ARS (Beyotime, China) at ambient temperature. Post staining, slides were rinsed three times with deionized water and subsequently observed under an optical microscope.

### 2.11. Western Blot Analysis

With connective tissues being carefully cleared away, fresh femur bone specimens were ground into powder using liquid nitrogen in a mortar. Radioimmunoprecipitation Assay (RIPA) buffer was adopted to lyse the bone powder on ice for the whole protein extraction. Subsequently, after a complete homogenization with a tissue grinder (10,000 rpm), the bone powder received 10 min of centrifugation at 10,000× *g* at 4 °C. We collected the resulting supernatant containing the extracted proteins as the analytical sample and maintained it at −80 °C for ≤4 weeks. The separated proteins after 12% SDS-polyacrylamide gel electrophoresis (Beyotime) were moved onto PVDF membranes (0.22 µm, Merck Millipore, Burlington, MA, USA) under electrotransfer. The membranes then received 12 min of blockage in 10% skim milk at room temperature. The membranes, post overnight incubation with primary antibodies, underwent three wash cycles with TBST (NCM Biotech), followed by 60 min culture with corresponding secondary antibodies (Beyotime, Nantong, China). Protein bands were visualized via ECL reagent (Epizyme, Sigma-Aldrich). The primary antibodies enlisted were NLRP3, ASC, caspase-1, IL-1β, IL-18, Beclin1, LC3, CTSD, VPS34, p62, and GAPDH. The employed secondary antibody was horseradish peroxidase-conjugated Goat anti-rabbit IgG. Densitometric evaluations of the resultant bands were performed, with normalization against GAPDH expression levels. Image J Program Analyzer (Media Cybernetics, Rockville, MD, USA) facilitated the quantitative analysis.

### 2.12. Statistical Analysis

All experiments were repeated independently no less than 3 times. Data are in the form of mean ± standard deviation (SD). One-way or two-way analysis of variance (ANOVA) followed by Tukey’s multiple comparisons test served for evaluating the between-group variance. A two-tailed Student’s *t*-test served for comparing two datasets in terms of their statistical significance. Statistical analyses relied on GraphPad Prism 9 software, and * *p* < 0.05, ** *p* < 0.01, and *** *p* < 0.001 denoted statistical significance.

## 3. Results

### 3.1. Effects of Trehalose on Body Weight and Bone Tissue Microstructure in Osteoporotic Rats Induced by Ovariectomy

**Figure 1:**
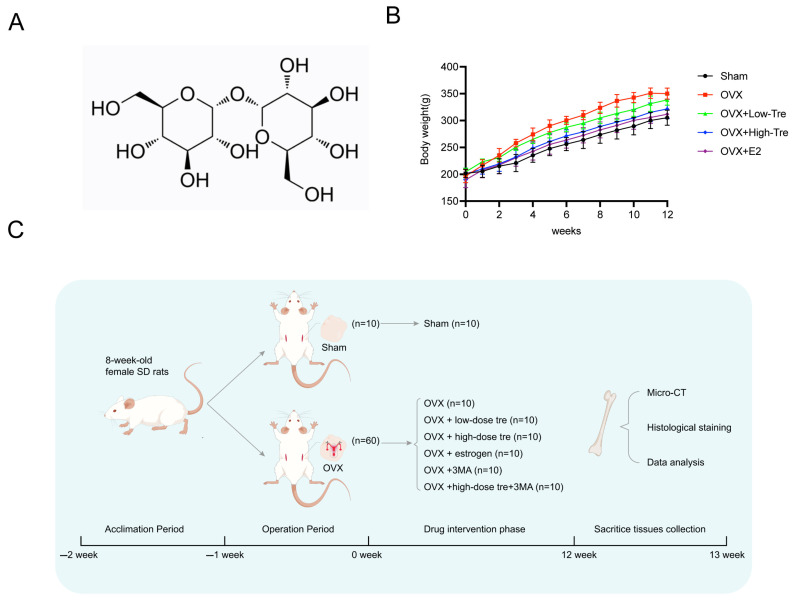
Trehalose alleviates bone deterioration caused by ovariectomy in rats. (**A**). Illustration of the molecular structure of trehalose. (**B**). Chart depicting the weight progression of rats across different groups over time. (**C**). Flow diagram outlining the steps and timeline of the in vivo experimental procedures.

**Figure 2:**
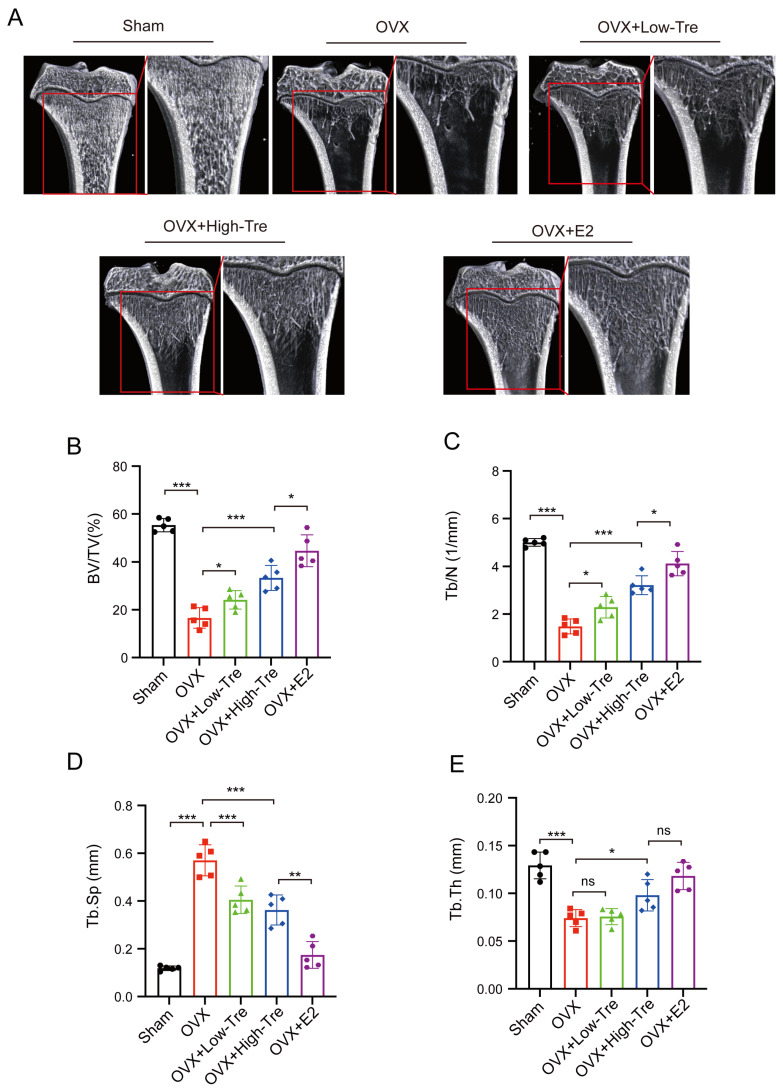
Trehalose displays protective effects on trabecular bone microstructure. (**A**). Micro-CT (μCT) images of the proximal tibia sections from each group. (**B**–**E**). Bar graphs representing quantitative analysis of specific bone microstructural parameters: B: BV/TV (%). C. Tb. N (1/mm). D. Tb. Sp (mm). (**E**). Tb. Th (mm). Each bar in the graphs represents the average value (mean) of the specific parameter ± its standard deviation (SD). ns: no significance, * *p* < 0.05, ** *p* < 0.01, and *** *p* < 0.001 indicated significance difference in statistical level when compared to the OVX group. Each experimental group consisted of 5 rats (n = 5).

A rat model of osteoporosis was generated via ovariectomy, followed by trehalose and estrogen treatment in the OVX rats (Figure 1A). Specifically, the trehalose treatment group were intraperitoneally injected with 1 g/kg and 2 g/kg trehalose on a daily basis for 12 weeks, and the positive control group was administered 200 μg/kg of estrogen. As illustrated in Figure 1B, a progressive increase in body weight was observed with aging. After the second week, the other groups showed a different body weight to that of the Sham group (*p* < 0.05) (Figure 1B). The comprehensive in vivo experimental design is outlined in Figure 1C. Employing three-dimensional (3D) image reconstruction, we discerned that bone trabeculae in the sham group exhibited a uniform distribution, systematic alignment, and interconnectedness, forming a lattice-like structure. Conversely, trabeculae in the OVX group appeared thin and sporadically placed, with significant reductions in both number and density (Figure 2A). After trehalose and estrogentreatment, bone microstructural parameters of BV/TV, Tb. Th, Tb. N and Tb. Sp were reversed (Figure 2B–E). In summary, trehalose treatment effectively prevented bone loss in OVX rats’ proximal tibia, positioning it as a potential therapeutic option.

### 3.2. Effect of Trehalose Treatment on Tibia Histomorphology in OVX Osteoporotic Rats

**Figure 3:**
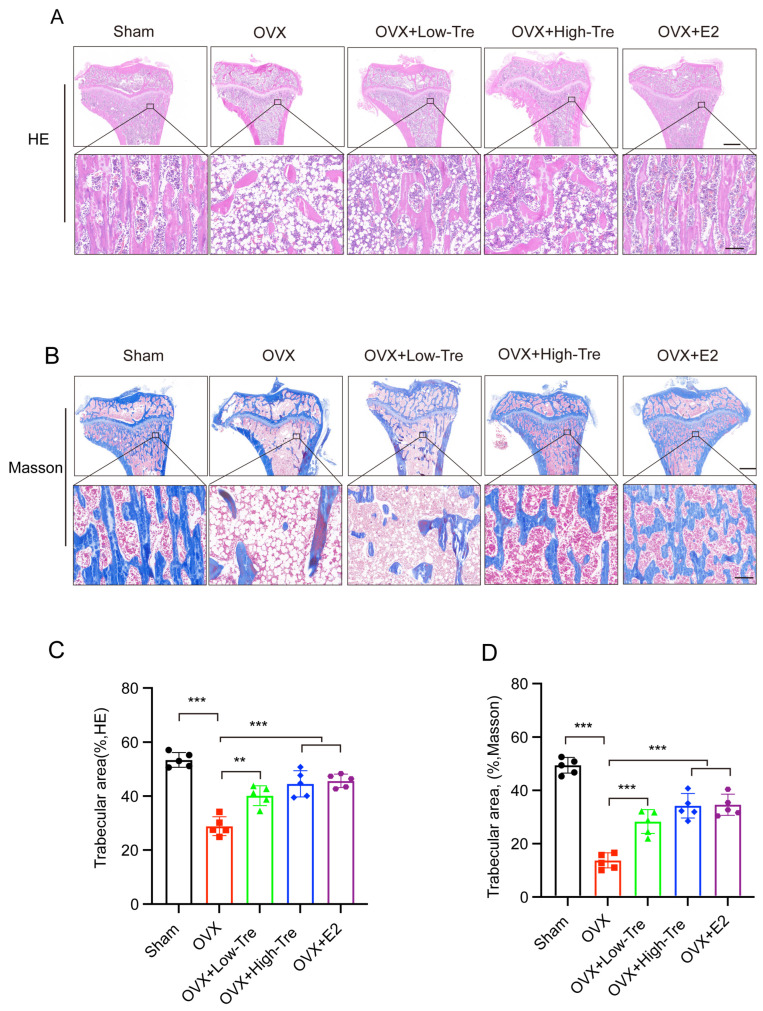
Histological analysis of trehalose influence on proximal tibial morphology in OVX-induced osteoporotic rats. (**A**) HE staining of proximal tibial tissue sections. The inset in the bottom panel highlights the enlarged regions. Scale bar: upper panel, 500 µm; bottom panel, 100 µm. n = 5. (**B**) Proximal tibial tissue sections visualized through Masson’s trichrome staining. The inset in the bottom panel showcases the magnified regions. Scale bar: upper panel, 500 µm; bottom panel, 100 µm. n = 5. (**C**,**D**) Analysis of trabecular area percentages by Image Pro Plus 6.0 software. Results are depicted as mean ± SD (n = 5). ** *p* <0.01 and *** *p* <0.001.

In Figure 3A,B, histological assessments of the rat proximal tibia are presented, with samples stained using hematoxylin and eosin (HE) and Masson’s trichrome, respectively. A comparative evaluation between the OVX and sham groups revealed pronounced osteoporotic alterations in the OVX cohort, as evidenced by a compromised trabecular network, attenuated and irregular bone trabeculae, and augmented trabecular spaces. Contrastingly, the trabecular morphology in the OVX + trehalose cohort displayed significant amelioration, typified by robust trabeculae and constricted trabecular voids. A computational quantification of trabecular bone area, leveraging ImageJ software (version number v1.8.0) on HE and Masson-processed samples, indicated a discernible reduction in the OVX group (28.85 ± 3.50) relative to the sham group (53.44 ± 2.71) (*p* < 0.001). In contrast, both the low-dose and high-dose trehalose groups presented a notable increase in trabecular bone area versus the OVX group, with the OVX + E2 group showing even better effects (45.66 ± 2.47), as demonstrated in Figure 3C,D. All these confirmed the inhibitory impact of trehalose and E2 on bone loss.

### 3.3. Trehalose Inhibits Pyroptosis through Suppression of NLRP3 Inflammasome Activation

**Figure 4:**
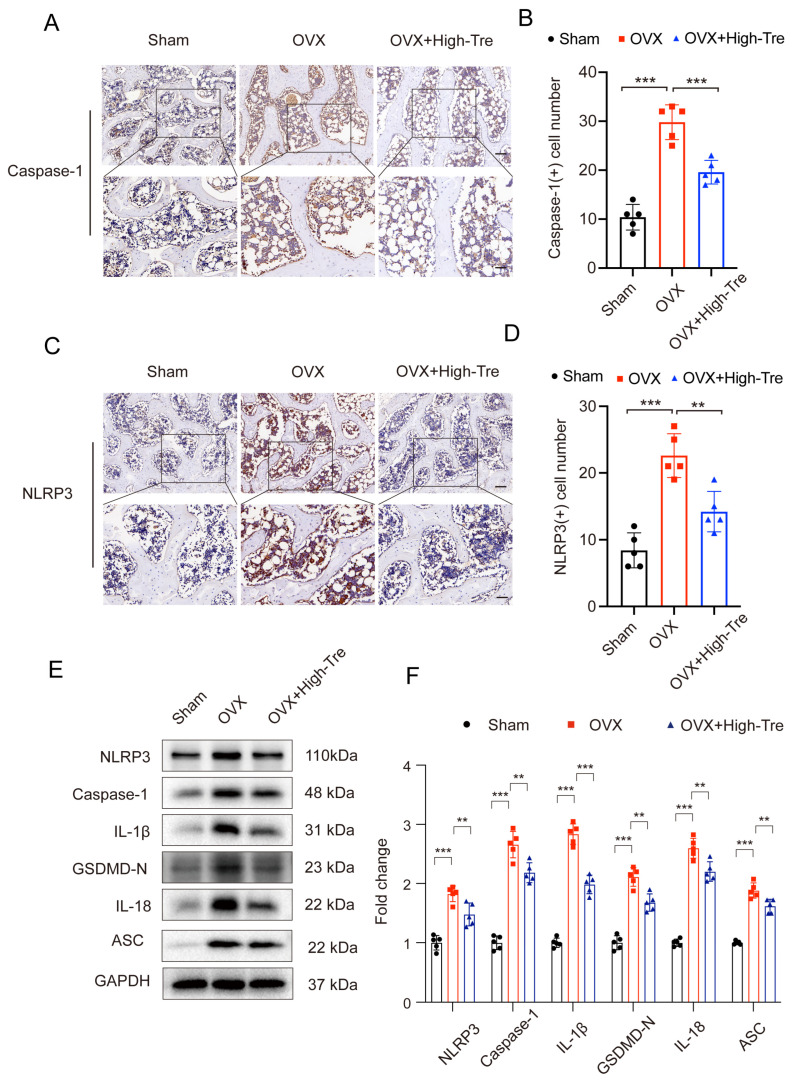
Trehalose mitigates osteoblastic pyroptosis. (**A**,**C**). IHC visualization of caspase-1 and NLRP3 expressions in decalcified bone samples. Each scale bar is equivalent to 50 µm. (**B**,**D**). Comparative analyses of caspase-1 and NLRP3 positive cell numbers across sham, OVX, and OVX + High-Tre group. Sample size is n = 5 for every group. (**E**,**F**). Protein expression patterns regarding NLRP3, Caspase-1, IL-1β, GSDMD-N, IL-18, and ASC are captured via Western blotting, followed by their respective quantitative assessments. Each group comprises a sample size of n = 5. Data presentation follows the mean ± SD format and ** *p* < 0.01, and *** *p* < 0.001 reported statistical significance.

The prototypical mechanism for pyroptosis initiation revolves around Caspase-1 activation, steered by the NLRP3 inflammasome. NLRP3 orchestrates the amalgamation of the adaptor molecule, ASC, and caspase-1. Due to this synergy, the conversion of pro-caspase-1 into active caspase-1 activates IL-1β and GSDMD. Intrigued by trehalose’s anti-inflammatory attributes, we investigated its potential interplay with the NLRP3 inflammasome concerning its anti-pyroptotic capacity. According to iImmunohistochemical staining, the density of NLRP3 and caspase-1 positive cells in proximal tibia presented a marked elevation in OVX group, which, however, was reduced in the OVX + High-Tre group (Figure 4A–D). Western blotting revealed identical expression alternations of NLRP3, caspase-1 to the immunohistochemical staining. The changes in GSDMD-N, IL-18, IL-1β and ASC were similar to NLRP3, caspase-1. These findings confirmed that Trehalose decreases the levels of pyroptosis-related biomarkers and thus inhibits pyroptosis after OVX (Figure 4E,F).

**Figure 5:**
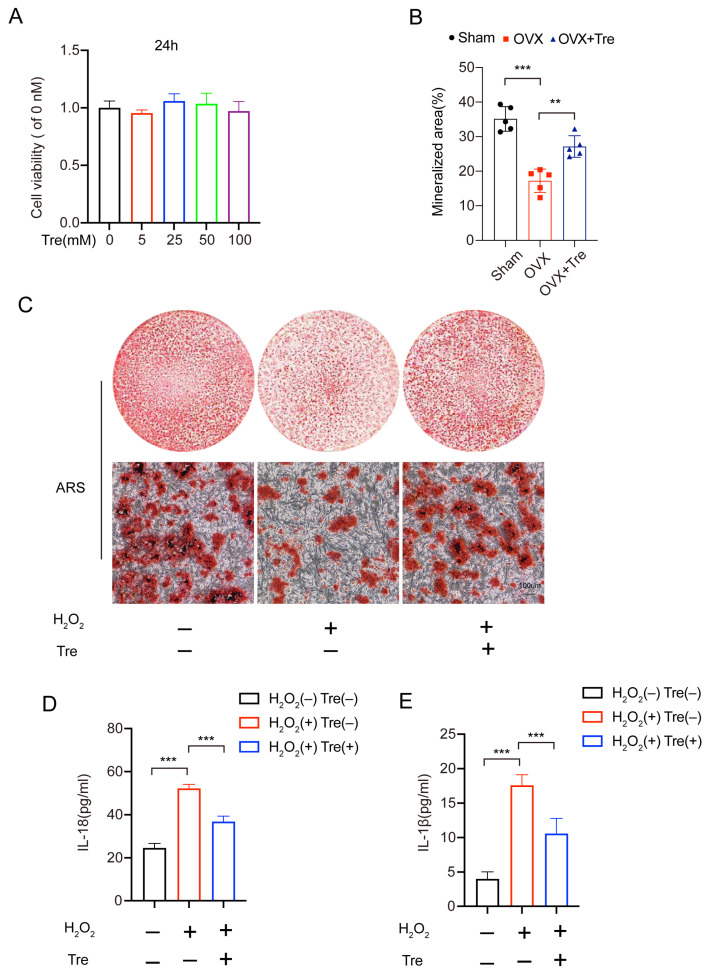
Trehalose reverses the adverse effects of H_2_O_2_ on osteoblasts. (**A**). CCK8 assays measuring the concentration-based effect of trehalose on osteoblast growth following 24 h processing (**B**,**C**). The effect of 25mM trehalose on osteogenic functions and mineral deposition under ARS staining. Scale bar: 100 µm. n = 5. Post-staining, the mineralized regions were quantified. n = 5. (**D**,**E**). ELISA detection of IL-1β and IL-18 in three groups of cell culture supernatants (n = 5). ** *p* < 0.01, and *** *p* < 0.001.

A CCK8 assay determined the cytotoxic effects of trehalose on osteoblast. No obvious cytotoxicity was detected at concentrations of 100 mM after 24 h of trehalose treatment. Actually, cell proliferation increased when cells were exposed to 25 mM for 24 h (Figure 5A). Based on the above results, we chose 25 mM trehalose as the most suitable concentration, and 25 mM trehalose was used in subsequent vitro experiments. The study adopted ARS staining to test the matrix mineralization after 14 days of trehalose-induced osteoblast differentiation, so as to ascertain the corresponding biological mechanisms of trehalose. Figure 5B,C illustrate the enhanced matrix mineralization due to trehalose treatment. In addition, we performed an ELISA test on the osteoblast, finding lower contents of IL-1β and IL-18 released into the supernatant in the trehalose-pretreated group compared with the H_2_O_2_ treatment (Figure 5D,E). Summarily, our findings advocate that trehalose suppresses osteoblastic pyroptosis.

### 3.4. Trehalose Promotes Autophagy

**Figure 6:**
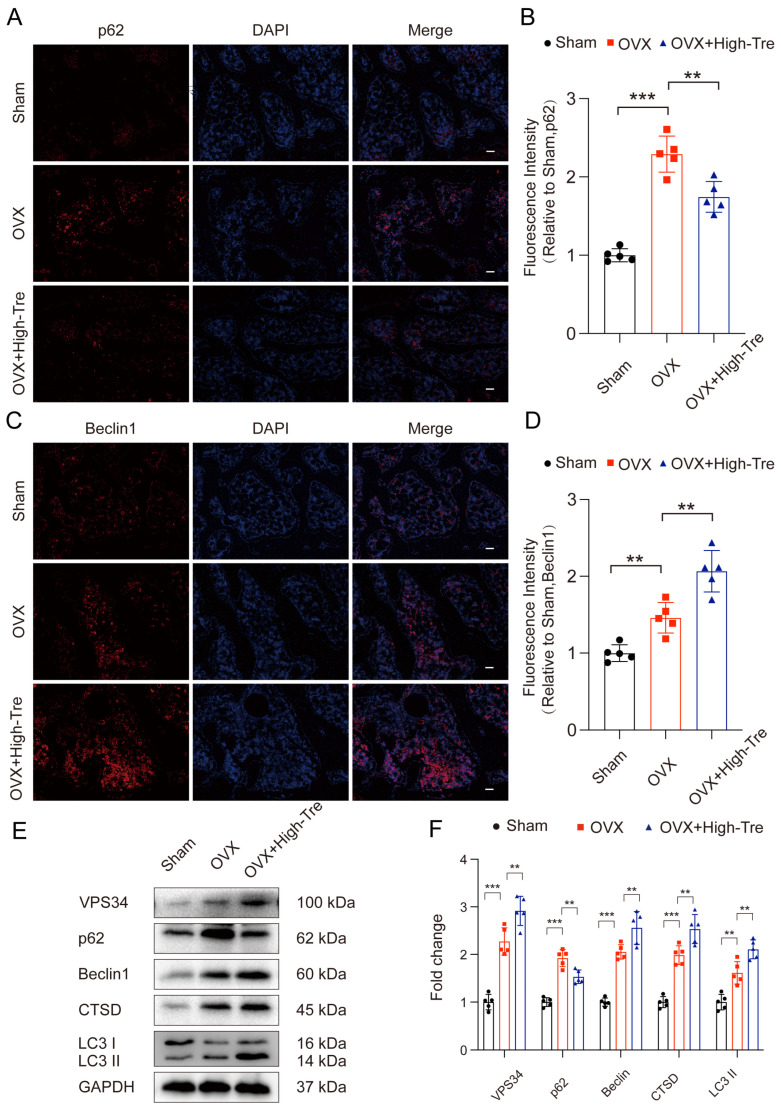
Trehalose induces autophagy. (**A**–**D**) Proximal tibial sections subjected to immunofluorescence labeling highlight p62 and Beclin1 distribution. Scale bar is set at 100 µm. (**E**) VPS34, P62, Beclin1, CTSD, and LC3II/LC3I expressions across the three examined groups are depicted through Western blotting. (**F**) Quantitative evaluations of the optical densities corresponding to VPS34, P62, Beclin1, CTSD, and LC3II/LC3I for each group. n = 5. ** *p* < 0.01, and *** *p* < 0.001 reported statistical significance.

To evaluate autophagic activity following OVX, immunofluorescence and Western blotting served for measuring the expression of key autophagy markers. Beclin-1, a critical molecule in autophagosome formation, promotes other autophagy-related proteins to be recruited to the phagophore so as to regulate the formation and maturation with regard to autophagosomes, with its expression increasing during autophagy. p62, a ubiquitin-binding protein, is one of the markers reflecting autophagic activity. The autophagy process is accompanied by the binding of p62 to ubiquitinated proteins, and a complex is formed by p62 and LC3-II protein. Localized to the autophagosome’s inner membrane, this complex is subsequently degraded within the autolysosome. When autophagic activity is reduced or impaired, p62 accumulates in the cytoplasm. Since the OVX + High-Tre group demonstrated superior outcomes, a dose of 2 mg/kg was adopted in subsequent experiments. Immunofluorescence staining revealed that the OVX group presented an obviously elevated p62 density in the proximal tibia, whereas the OVX + Tre group showed a comparatively lower p62 density (Figure 6A,B). The OVX group had elevated Beclin1 puncta expression in the proximal tibia versus the sham group. Moreover, the OVX + Tre group had remarkably higher Beclin1 puncta expression versus the untreated OVX group (Figure 6C,D). Western blot analysis demonstrated the upregulation of autophagy initiation markers, including VPS34, Beclin1, CTSD, and the LC3II/LC3I ratio, and reduced p62 levels following trehalose treatment. These results indicate that trehalose activated autophagic flux (Figure 6E,F). In conclusion, our data demonstrate that trehalose enhanced the expression of autophagic lysosome- and autophagosome-related biomarkers and lowered the levels of substrate proteins, thereby improving autophagic flux after OVX.

### 3.5. Inhibiting Autophagy Reverses the Inhibitory Effect of Trehalose on Pyroptosis

**Figure 7:**
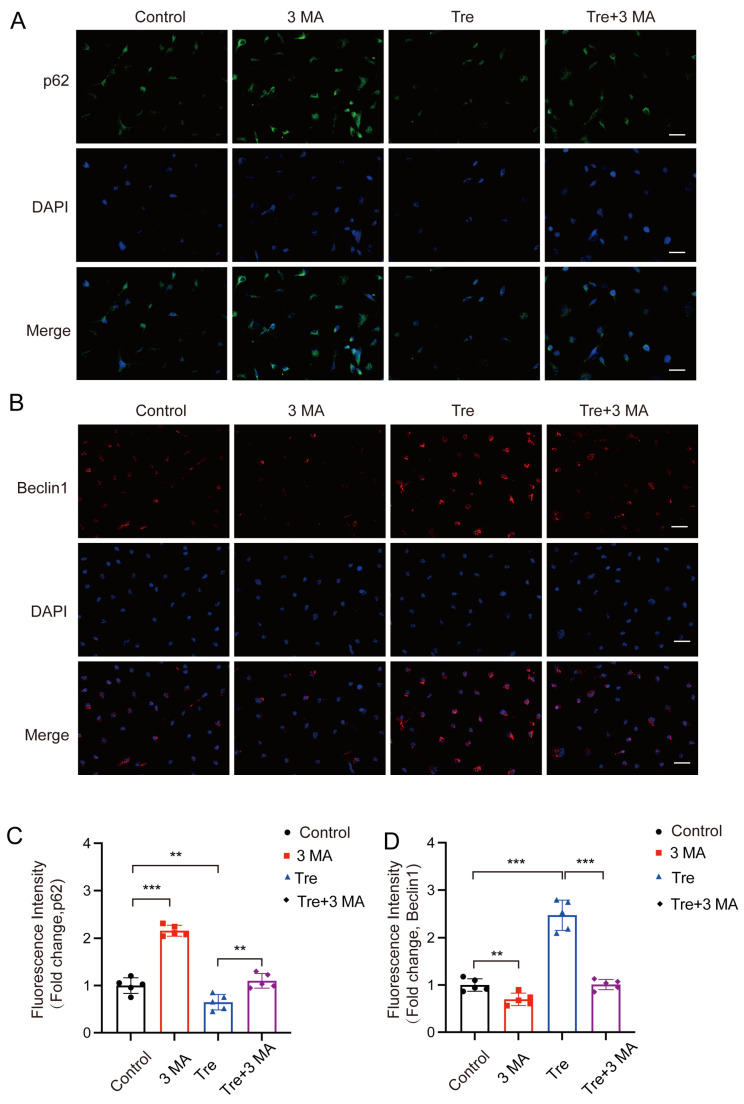
Inhibiting autophagy reverses the effect of inhibiting trehalose on pyroptosis in vitro. (**A**,**B**) Diagram illustrating that BMSCs were treated with 3-MA (2.5 mM) for two hours after trehalose treatment (25 μM, 2 h). Beclin1 and P62 level analysis by immunostaining. (**C**,**D**) Quantitative analysis of Beclin1 and P62 levels in cells. scale bar: 50 μm. ** *p* < 0.01; *** *p* < 0.001, n = 5.

Autophagy inhibitor 3 MA is used in conjunction with trehalose to evaluate if trehalose’s inhibition of pyroptosis is due to the activation of autophagy. Initially, we confirmed the impact of 3 MA on autophagy in osteoblasts through immunofluorescence analysis. Relative to the control group, the 3 MA group exhibited enhanced p62 density and decreased Beclin1 signaling, whereas the trehalose group exhibited reduced p62 density and significantly increased Beclin1 signaling. However, the combined treatment of 3 MA and trehalose led to increased p62 density and significantly reduced Beclin1 signaling. This suggests that 3 MA effectively inhibits the activation of autophagy by trehalose (Figure 7A–D).

**Figure 8:**
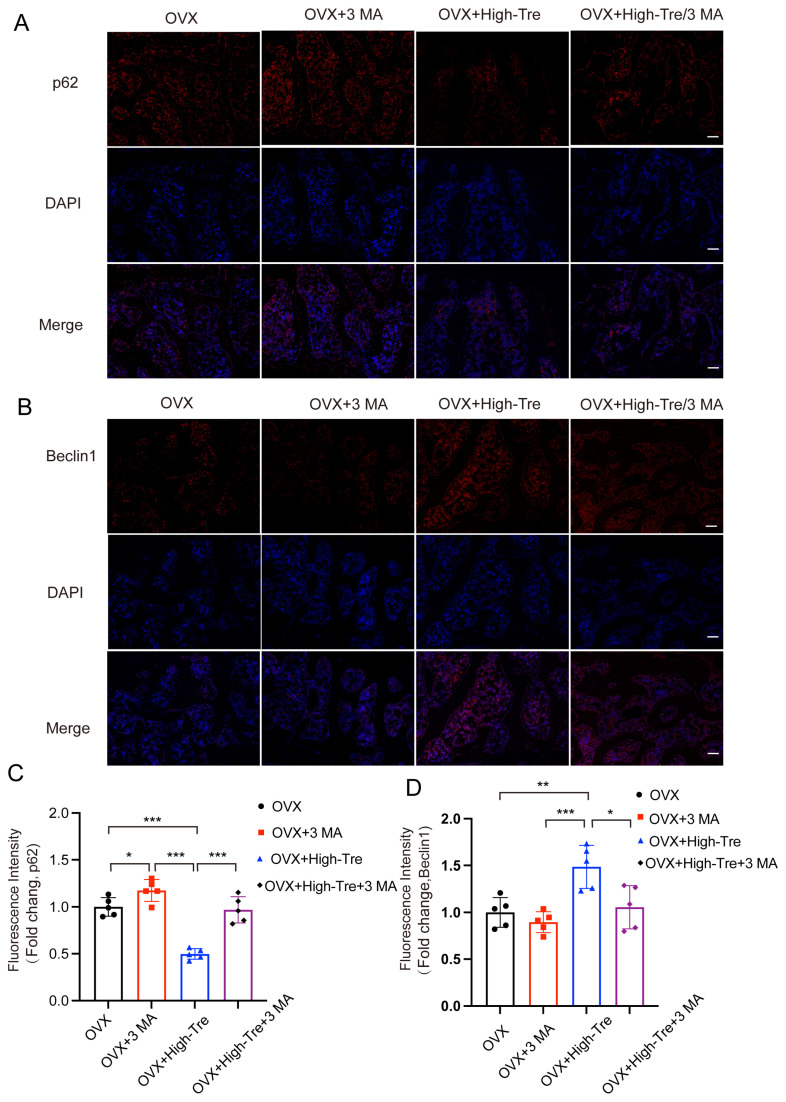
Inhibition of autophagy reverses the suppressive effect of trehalose on pyroptosis in vivo. (**A**,**B**) Immunofluorescence analysis of proximal tibial sections revealed the expression patterns of p62 and Beclin1 (scale bar = 100 μm). (**C**,**D**) Quantitative assessment of Beclin1 and p62 levels in the proximal tibia. Data presentation follows the mean ± SD format and * *p* < 0.05, ** *p* < 0.01, *** *p* < 0.001 (n = 5) reported statistical significance.

**Figure 9:**
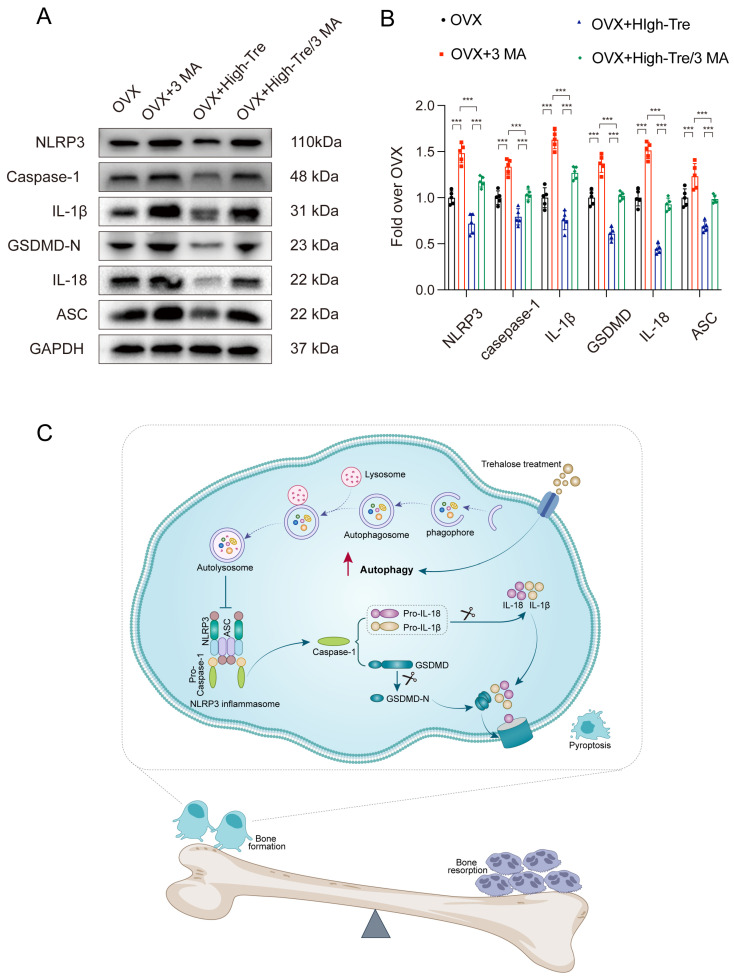
Inhibition of autophagy reverses the suppressive effect of trehalose on pyroptosis in vivo. (**A**) Western blot analysis assessing IL-18, IL-1β, GSDMD, Caspase-1, ASC, and NLRP3 expressions across the different experimental groups. (**B**) Quantitative evaluation of the OD for NLRP3, Caspase-1, GSDMD-N, IL-18, IL-1β, and ASC for each group. Data presentation follows the mean ± SD format (n = 5). *** *p* < 0.001 reported statistical significance. (**C**) A schematic diagram demonstrating the protective mechanism of trehalose in estrogen-deficient osteoporosis. Trehalose promotes autophagy in osteoblasts, thereby attenuating inflammasome activation. This leads to the inhibition of osteoblast pyroptosis and the associated pro-inflammatory responses. The enhanced survival and functionality of osteoblasts contribute to a shift in the bone remodeling balance towards bone formation, ultimately counteracting osteoporosis.

Subsequently, the autophagic and pyroptotic activities in animals treated with trehalose and co-administered 3 MA were assessed through immunofluorescence staining and Western blotting. Immunofluorescence of animal tissue sections showed that trehalose enhanced autophagy, but this enhancement was reversed when co-administered with 3 MA, conforming to the cellular experimental results (Figure 8A–D). Western blot analysis examined the ASC, GSDMD-N, Caspase-1, NLRP3, IL-1β, and IL-18 expressions in bone tissues. The results indicated that the trehalose + 3MA group presented higher optical density (OD) values for ASC, Caspase-1, GSDMD-N, IL-1β, IL-18, and NLRP3 versus the trehalose group alone (Figure 9A,B). Taken together, the co-administration of 3 MA with trehalose weakens the effect of trehalose in reducing pyroptosis, implying that the enhancement of autophagy by trehalose might be its mechanism for inhibiting pyroptosis.

## 4. Discussion

In recent years, researchers have made notable strides in understanding osteoporosis pathogenesis. Increasing research suggests that trehalose may serve both as a potential biomarker and treatment for osteoporosis. However, experimental data on trehalose’s role in osteoporosis, especially its bone-protective effects via pyroptosis regulation, remain scarce. Our findings suggest that trehalose activates autophagy and inhibits pyroptosis, preventing OVX-induced bone loss as well as ameliorating bone metabolism. Mechanically, estrogen deficiency leads to the development of osteoblast pyroptosis, while the activation of autophagy can inhibit the occurrence of pyroptosis. Here, we determined that trehalose alleviates estrogen deficiency induced osteoporosis by enhancing autophagy and inhibiting pyroptosis (as depicted in Figure 9C).

Inflammation is recognized as a key contributor to osteoporosis. Numerous cytokines and chemokines, including TNF-α, IL-1β, and IL-18, are implicated in bone resorption processes. Chronically increased TNF-α levels increase the risks of inflammatory-related diseases like osteoporosis [30,31]. TNF-α promotes osteoclast differentiation by virtue of the nuclear factor kappa-B (NF-κB) pathway, upregulating genes such as RANK, while simultaneously inhibiting osteoblast differentiation by suppressing osteogenic markers like RUNX2 [32,33]. IL-1β has a dual role in bone metabolism. It acts as a stimulator of bone resorption and, at elevated concentrations, inhibits osteogenic differentiation. IL-1β is known to enhance RANKL expression in osteoblasts and bone marrow mesenchymal stem cells, assisting osteoclast development [34]. Moreover, IL-1β, upon binding to its receptors on T lymphocytes, B lymphocytes, and macrophages, promotes RANKL production, facilitating osteoclast differentiation and activation [35,36]. In high doses, IL-1β suppresses osteogenic differentiation through NF-κB activation, which interrupts the BMP/Smad signaling pathway [37]. Additionally, IL-1β can downregulate Runx2, essential for osteoblastic differentiation, via the MAPK pathway [38]. IL-18, closely related to IL-1β structurally, promotes osteoclastogenesis by upregulating IFN-γ production in the inflammatory setting caused by pyroptosis. Directly, IL-18 can influence T lymphocytes to support bone resorption [39]. Based on the above mechanisms, trehalose has anti-inflammatory properties that can possibly prevent deeper bone loss for females. However, the specific mechanism of action of trehalose on PMOP remains unclear.

Pyroptosis is a specific form of PCD, and numerous inflammatory cytokines are released in the process, notably IL-1β, and IL-18 [40]. Central to pyroptosis is the inflammasome, an inflammation-inducing protein complex found in various cell types. NLRP3 is an inflammasome the most extensively examined, assembling with ASC and pro-caspase-1 in response to cellular stimuli from PAMPs or DAMPs, subsequently promoting IL-1β to be maturated and released through activated caspase-1 [41]. This activation process upregulates pyroptosis markers, including caspase-1, IL-1β, and IL-18, inducing cellular pore formation and ultimately leading to pyroptosis. Numerous studies examined the relevance of NLRP3 to osteoporosis, indicating that its inhibition can mitigate osteoporosis, cell death, and inflammation [6,17]. Trehalose, a renowned natural disaccharide, has been shown to mitigate myocardial ischemia/reperfusion injury by inhibiting pyroptosis [42]. Our study engaged in assessing osteoblast pyroptosis and the role of trehalose in cellular and animal models, and found that the expression of pyroptosis related proteins was increased in OVX rat bone tissue, indicating that pyroptosis occurred in OVX rat osteoblasts. In vitro experiments ascertained the decreased expressions of these proteins in osteoblasts treated with trehalose. Building upon this foundation, estrogen-deficiency-induced osteoporosis may experience osteoblast pyroptosis, and trehalose therapy is important for reducing osteoblast pyroptosis.

Autophagy is an intracellular degradation pathway that targets damaged organelles, cellular macromolecules, and protein aggregates for lysosomal breakdown [43]. Dysregulated autophagy has been implicated in the excessive secretion of IL-1β and IL-18 [44]. Numerous studies suggest that autophagy can dampen inflammasome activity, mitigating inflammatory responses and pyroptosis [45]. Recent findings highlight the activation of autophagy in osteoporosis, postulating its adaptive role in promoting cell survival [46,47]. Trehalose, a non-reducing disaccharide organisms of bacteria, fungi, plants, and invertebrates, etc., is composed of two d-glucose units connected by an α-1,1 linkage [48]. Beyond its biological occurrence, trehalose is recognized as an inducer of mTOR-independent autophagy [49,50]. Recent research underscores the potential of trehalose in osteoporosis prevention [27]. Specifically, trehalose has been shown to counteract palmitic acid-induced osteoblast apoptosis by modulating autophagy through the AMPK/mTOR/ULK1 pathway [28]. In addition, trehalose has demonstrated its ability to enhance bone mass in cirrhotic rats, suggesting the pivotal role of autophagy in trehalose-mediated osteoporosis treatment [29]. Subsequently, we evaluated the effect of trehalose on osteoblast autophagy in both cellular and animal models. Our results suggest that trehalose mitigated estrogen-deficiency-induced osteoporosis and the inhibition of bone formation induced by H_2_O_2_ in osteoblasts. According to mechanism studies, trehalose significantly activates autophagy, thereby inhibiting pyroptosis in osteoblasts. When autophagy was inhibited with 3-MA, the therapeutic effect of trehalose was reversed.

To sum up, we delved into the interplay between trehalose, autophagy, and pyroptosis, using an ovariectomized rat model treated with trehalose. We found that trehalose augments autophagy in OVX rats. Enhanced autophagy, in turn, restricts inflammasome activation, leading to pyroptosis inhibition (Figure 9C). Collectively, trehalose’s influence leads to better outcomes in osteoporosis These findings offer groundbreaking preclinical evidence for trehalose’s therapeutic potential in osteoporosis, paving the way for innovative treatments for osteoporosis and related skeletal disorders.

## 5. Conclusions

In summary, our results demonstrated that trehalose suppressed osteoblastic pyroptosis and OVX-mediated bone loss in vitro and in vivo. Mechanistically, trehalose promoted autophagy, thereby inhibiting pyroptosis. Given these insights, trehalose emerges as a promising therapeutic candidate for osteoporosis treatment. However, considering the unclear molecular mechanism underlying the way trehalose promotes autophagy and suppresses pyroptosis in osteoblasts, future researches are suggested to assess the feasibility and safety of trehalose in clinical settings in the long run, as well as to explore its potential synergistic effects with other therapeutic agents to optimize osteoporosis treatment strategies.

## Data Availability

The original contributions presented in the study are included in the article, further inquiries can be directed to the corresponding author.
